# Association of Glaucoma with the Risk of Peripheral Arterial Occlusive Disease: A Retrospective Population-Based Cohort Study

**DOI:** 10.3390/jcm12144800

**Published:** 2023-07-20

**Authors:** Han-Wei Yeh, Chi-Tzu Chung, Chao-Kai Chang, Chao-Bin Yeh, Bo-Yuan Wang, Chia-Yi Lee, Yu-Hsun Wang, Liang-Tsai Yeh, Shun-Fa Yang

**Affiliations:** 1 School of Medicine, Chang Gung University, Taoyuan City 333, Taiwan; 2 Department of Medical Education, Chang Gung Memorial Hospital, Linkou, Taoyuan City 333, Taiwan; 3 Department of Emergency Medicine, School of Medicine, Chung Shan Medical University, Taichung 402, Taiwan; 4 Department of Emergency Medicine, Chung Shan Medical University Hospital, Taichung 402, Taiwan; 5 Institute of Medicine, Chung Shan Medical University, Taichung 402, Taiwan; 6 Nobel Eye Institute, Taipei 100, Taiwan; 7 Department of Optometry, Da-Yeh University, Chunghua 515, Taiwan; 8 Department of Ophthalmology, Jen-Ai Hospital Dali Branch, Taichung 412, Taiwan; 9 Department of Medical Research, Chung Shan Medical University Hospital, Taichung 402, Taiwan; 10 Department of Anesthesiology, Changhua Christian Hospital, Changhua 500, Taiwan; 11 Department of Post-Baccalaureate Medicine, College of Medicine, National Chung Hsing University, Taichung 402, Taiwan

**Keywords:** glaucoma, peripheral arterial occlusive disease, match cohort, risk

## Abstract

This study aimed to investigate the potential association between glaucoma and peripheral arterial occlusive disease. The study recruited patients, including 101,309 with glaucoma and 1,860,528 without a glaucoma diagnosis, from a population of 2 million patients in the Longitudinal Health Insurance Database. Propensity score matching was performed between the two groups, matching for age, sex, and comorbidities. In total, 95,575 patients with glaucoma and 95,575 patients without glaucoma were analyzed for their risk of developing peripheral arterial occlusive disease. The analysis of the data revealed that the glaucoma group had a higher incidence density (ID = 4.13) of peripheral arterial occlusive disease than the non-glaucoma group (ID = 3.42). The relative risk for the glaucoma group was 1.21 (95% C.I. = 1.15–1.28). Cox proportional hazard model analysis indicated that the glaucoma group had a higher risk of developing peripheral arterial occlusive disease (HR = 1.18; 95% C.I. = 1.12–1.25). The subgroup analysis of the risk of PAOD showed that the glaucoma group had a higher risk of developing peripheral arterial occlusive disease in the age group of 20 to 39 (*p* for interaction = 0.002). In conclusion, patients with glaucoma were associated with a higher risk of subsequent peripheral arterial occlusive disease compared with those without a diagnosis of glaucoma.

## 1. Introduction

Glaucoma is a group of eye diseases that damage the optic nerve and may lead to visual field loss and ultimately irreversible blindness if left untreated [1]. The etiology is not fully characterized; the main cause of glaucoma is traditionally thought to be increased intraocular pressure (IOP) [2]. However, glaucoma is more accurately defined as an optic neuropathy involving characteristic atrophy of the optic nerve head, often accompanied with typical visual field defects [3]. It is related to progressive degeneration and loss of retinal ganglion cells (RGCs) and their axons [4]. Glaucoma is often, but not always, associated with increased intraocular pressure (IOP); eyes may have an IOP within the normal range and still develop glaucoma [5]. There are different types of glaucoma, generally categorized by the anterior chamber (iridocorneal) angle and the underlying etiology, including open-angle glaucoma (OAG), angle-closure glaucoma, mixed mechanism glaucoma, and secondary glaucoma. Even though the clear pathophysiological mechanism is still under investigation, the examination of a glaucomatous optic nerve reveals “cupping,” which looks like a “hollowing out” of the optic nerve head. Angle-closure glaucoma is more prevalent in Asian populations [6,7], while open-angle glaucoma is more common in European or African populations [8,9]. The number of people with glaucoma worldwide is predicted to increase to over 111 million in 2040 [10]. The risk factors for developing both primary angle-closure glaucoma and open-angle glaucoma include age, family history, and pseudoexfoliation [11,12].

Peripheral arterial occlusive disease (PAOD) is a clinical presentation of systemic atherosclerosis that is necessarily associated with coronary artery disease, carotid artery disease, and cerebrovascular diseases [13]. The artherosclerotic process causes arterial luminal narrowing due to the lipid or the fibrosis material that accumulates between the intimal and the medial layers of the tissue. It may be silent or present with a variety of symptoms and signs indicative of extremity ischemia. The most common presentation is intermittent claudication and pain; critical limb ischemia is characterized by severely decreased circulation, ischemia pain, ulceration, and may lead to limb loss or even death [14]. The worldwide prevalence of lower extremity peripheral artery disease is between 3% and 12% [15], rising to 20% of people over 75 years old [16]. The risk factors of PAOD include age, family history, smoking, diabetes, hypertension, hyperlipidemia, and chronic kidney disease (CKD), similar to those that promote the development of coronary heart disease [17,18,19,20].

Many publications have indicated that both PAOD and glaucoma could induce vascular changes [21]. Vascular factors have been suggested to play a role in glaucoma development, based on numerous studies showing associations of glaucoma with blood pressure, ocular perfusion pressure, vasospasm, cardiovascular disease, and ocular blood flow [22]. Systemic factors may also play a role, because there is some evidence that cardiac autonomic dysfunction, as measured by heart rate variability, may correlate with the presence of normal pressure glaucoma. However, few publications have mentioned the association of PAOD with glaucoma. In this study, we enrolled patients with glaucoma and without glaucoma from the Longitudinal Health Insurance Database 2000 (LHID 2000) to clarify the association of glaucoma and PAOD. We hypothesized that patients with glaucoma would have a significantly high risk of PAOD.

## 2. Materials and Methods

### 2.1. Data Sources

The longitudinal health insurance database was managed by the Health and Welfare Data Science Center (HWDC) in Taiwan. The database comprised two million beneficiaries that were randomly sampled from the 2000 registry for beneficiaries with the entire Taiwan population. The database contained medical claims, including drug medication, medical operation, procedure, and expenses in outpatients and inpatient care from 2000 to 2018. The diagnosis of disease was based on the International Classification of Diseases, 9th and 10th revisions, Clinical Modification (ICD-9-CM; ICD-10-CM). The study received approval from the ethical review board of the Chung Shan Medical University Hospital (CS1-20056).

### 2.2. Study Group and Outcome Measurement

This study was conducted using the retrospective cohort study design. A new diagnosis of glaucoma (ICD-9-CM = 364.22, 365; ICD-10-CM = H40, H42), except for drug-induced glaucoma (ICD-9-CM = 365.31, 365.32; ICD-10-CM = H40.6), and age greater than or equal to 20 years old, from 2002 to 2017, was defined as the glaucoma group. To verify the accuracy of the diagnoses, at least 3 outpatient visits or 1 hospitalization recommendation by an ophthalmologist were required. The index date was defined as the first diagnosis date of glaucoma. Additionally, we excluded diagnoses of peripheral arterial occlusive disease (PAOD, ICD-9-CM = 443.8, 443.9, 444; ICD-10-CM = I73.81, I73.89, I73.9, I74.01, I74.09, I74.11, I74.2, I74.3, I74.4, I74.5, I74.8, I74.9, I79.1, I79.8) before the index date to ensure the new-onset subjects. The non-glaucoma group was defined as never having a diagnosis of glaucoma (ICD-9-CM = 364.22, 365; ICD-10-CM = H40, H42) from 2000 to 2018.

The analytic outcome was defined as new-onset peripheral arterial occlusive disease (PAOD, ICD-9-CM = 443.8, 443.9, 444; ICD-10-CM = I73.81, I73.89, I73.9, I74.01, I74.09, I74.11, I74.2, I74.3, I74.4, I74.5, I74.8, I74.9, I79.1, I79.8). To verify the accuracy of the diagnoses, at least 3 outpatient visits or 1 hospitalization was required. Both groups were traced until the onset of PAOD, death, or 31 December 2018, whichever occurred first.

### 2.3. Covariates and Matching

The baseline characteristics were age, sex, and underlying comorbidities, including hypertension (ICD-9-CM = 401–405; ICD-10-CM = I10–I15), hyperlipidemia (ICD-9-CM = 272; ICD-10-CM = E78), chronic liver disease (ICD-9-CM = 571; ICD-10-CM = K70, K73, K74, K75.4, K75.81, K76.0, K76.89, K76.9), chronic kidney disease (ICD-9-CM = 585; ICD-10-CM =N184, N185, N186, N189), diabetes (ICD-9-CM = 250; ICD-10-CM = E10, E11, E13), chronic obstructive pulmonary disease (ICD-9-CM = 491, 492, 496; ICD-10-CM = J41–J44), ischemic heart disease (ICD-9-CM = 410–414; ICD-10-CM = I20–I25), stroke (ICD-9-CM = 433–438; ICD-10-CM = I63, I64), intracranial bleeding (ICD-9-CM = 430–432; ICD-10-CM = I60–I62), deep vein thrombosis (ICD-9-CM = 453.8; ICD-10-CM = I82.21, I82.29, I82.40, I82.41, I82.42, I82.43, I82.44, I82.49, I82.4Y, I82.4Z, I82.50, I82.51, I82.52, I82.53, I82.54, I82.59, I82.5Y, I82.5Z, I82.60, I82.61, I82.62, I82.70, I82.71, I82.72, I82.81, I82.89, I82.9, I82.A, I82.B, I82.C), varicose veins of lower extremities (ICD-9-CM = 454; ICD-10-CM = I83), and psoriasis (ICD-9-CM = 696; ICD-10-CM = L40, L41, L42, L44, L45, L30.5, L94.5). Those comorbidities were defined before the index date within one year and at least three outpatient visits or one hospitalization.

A 1:4 matching by age and sex was conducted to generate an index date for the comparison subjects that had synchronous initiation. Then, propensity score matching was performed by age, sex, and comorbidities. The propensity score was a probability from zero to one that was estimated through logistic regression. The glaucoma or the comparison group was the binary variable. By matching the propensity score, the heterogeneity in both group was harmonious.

### 2.4. Statistical Analysis

Comparisons of the glaucoma group and non-glaucoma group were performed using the absolute standardized differences (ASD). When the absolute standardized differences were less than 0.1, the divergence of both groups were small [23]. The Poisson regression model was used to calculate the relative risk (RR) between the glaucoma group and non-glaucoma group. Kaplan–Meier analysis was used to calculate the cumulative incidence of PAOD among the two groups; significance was assessed using the log-rank test. The multivariate Cox proportional hazard model was used to estimate the hazard ratios between glaucoma group and non-glaucoma group. SAS version 9.4 (SAS Institute Inc., Cary, NC, USA) was used for the statistical analysis.

## 3. Results

### 3.1. Characteristics of the Participants

**Figure 1:**
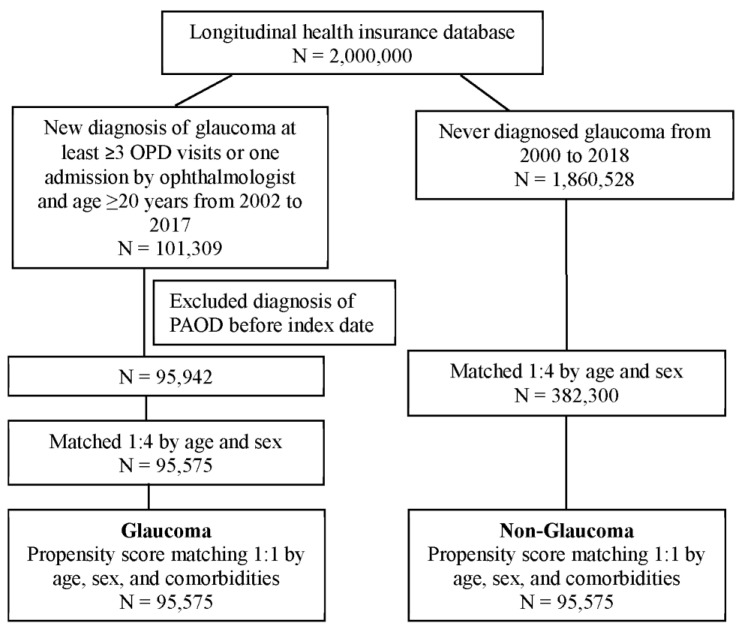
Flowchart of patient selection.

In our study, 101,309 patients with glaucoma and 1,860,528 of patients who had never been diagnosed with glaucoma were identified. After the patients who had been diagnosed with PAOD before the index date were excluded, 95,942 patients remained in the glaucoma cohorts. To analyze the risk of PAOD, both cohorts were conducted 1:1 with PSM by age, sex, and comorbidities. Finally, 95,575 patients in the glaucoma cohort and 95,575 patients without glaucoma on matched cohorts were analyzed for their risk of PAOD (Figure 1). Demographic characteristics of both study cohorts are shown in Table 1. The mean ages in the glaucoma and non-glaucoma cohorts were 55.0 and 55.5, respectively. The majority of patients were female (52%). After propensity score matching, all absolute standardized differences were less than 0.1. It appeared that the divergence in age, sex, and comorbidities between both groups was minor. The mean follow-up durations of glaucoma and non-glaucoma patients were 7.6 years (SD: 4.5 years) and 7.4 years (SD: 4.5 years), respectively. The average times to PAOD onset of glaucoma and non-glaucoma were 5.1 years (SD: 3.7 years) and 5.0 years (SD: 3.7 years), respectively.

### 3.2. Risk of PAOD in the Glaucoma and Non-Glaucoma Group

**Figure 2:**
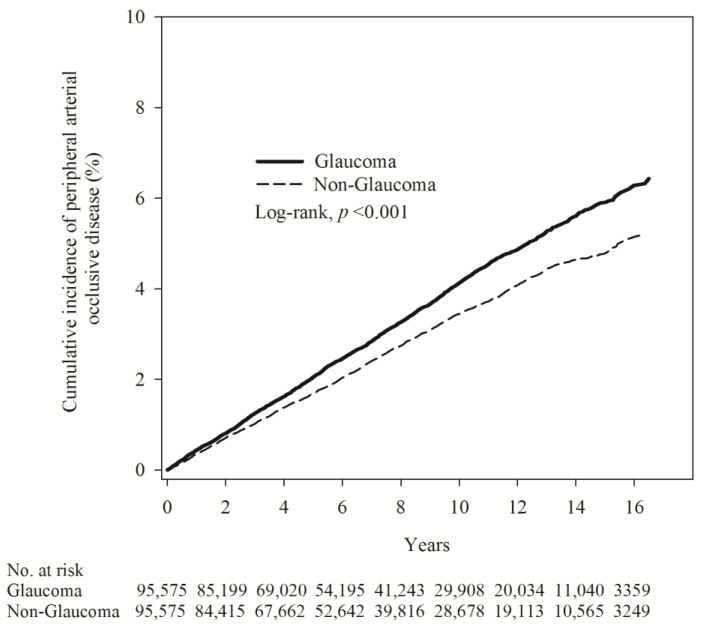
Kaplan–Meier curves showing the cumulative proportions of PAOD in patients with glaucoma and patients without glaucoma.

Next, Poisson regression was employed to compare the relative risk of glaucoma and non-glaucoma. The results indicated that the glaucoma group had a higher incidence density (ID = 4.13) of PAOD than the non-glaucoma group (ID = 3.42). The relative risk was 1.21 (95% C.I. = 1.15–1.28) (Table 2). The cumulative incidence of PAOD risk in both groups revealed that the risk of PAOD was higher in the glaucoma group than in the non-glaucoma group (log-rank test, *p* < 0.001) (Figure 2).

### 3.3. Analysis of the Risk of PAOD Using the Cox Proportional Hazard Model

Analyzing the risk of PAOD using the Cox proportional hazard model indicated that the glaucoma group had a higher PAOD risk (HR = 1.18; 95% C.I. = 1.12 to 1.25). The risk of PAOD was also higher among patients aged 40–64 years (HR =4.75; 95% C.I. = 4.03 to 5.61) and aged equal to or greater than 65 years (HR = 9.32; 95% CI = 7.88 to 11.01) compared with an age under 40 years. In addition, comorbidities such as hypertension, chronic kidney disease, diabetes, COPD, ischemic heart disease, stroke, and deep vein thrombosis were stronger risk factors of PAOD (Table 3).

### 3.4. Subgroup Analysis of the Risk of PAOD between the Glaucoma and Non-Glaucoma Group after PSM

**Figure 3:**
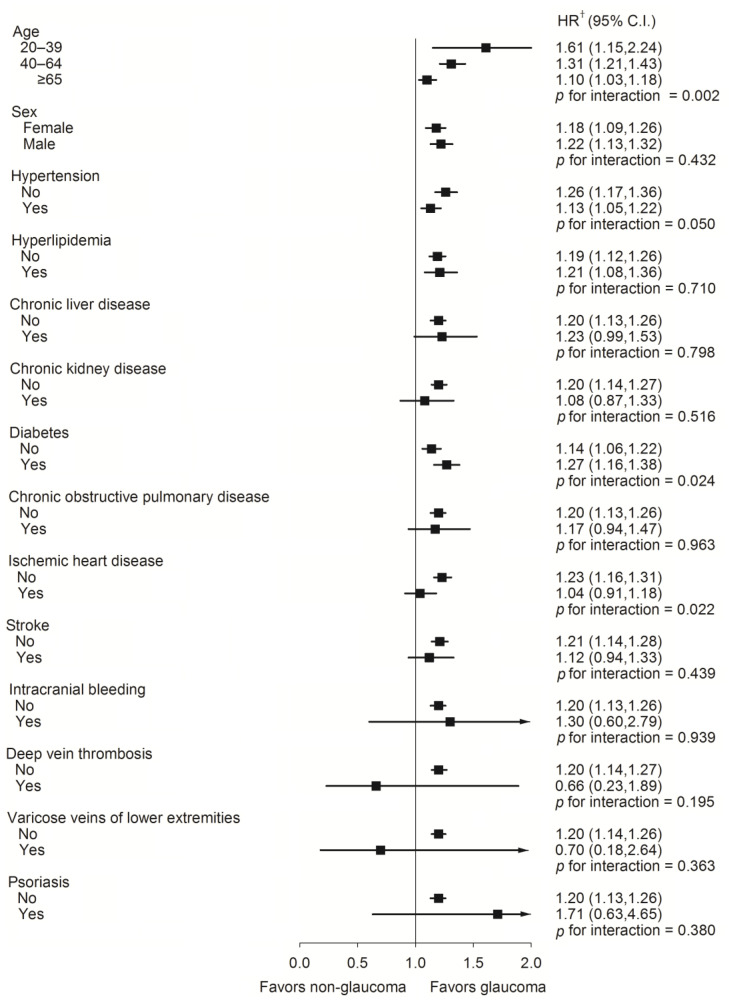
A forest plot analysis assessed the peripheral arterial occlusive disease risk rate between glaucoma and non-glaucoma individuals. † Adjusted for all variables. Adjusted for age, sex, hypertension, hyperlipidemia, chronic liver disease, chronic kidney disease, diabetes, chronic obstructive pulmonary disease, ischemic heart disease, stroke, intracranial bleeding, deep vein thrombosis, varicose veins of lower extremities, and psoriasis.

Furthermore, in the subgroup analysis of the risk of PAOD, the glaucoma group had a higher risk of PAOD compared with the non-glaucoma group among those aged 20 to 39 (*p* for interaction = 0.002). The glaucoma group also had higher risk of PAOD in the non-hypertension and non-ischemic heart disease groups (Figure 3).

## 4. Discussion

In this study, patients newly diagnosed with glaucoma and patients never diagnosed with glaucoma were enrolled to analyze their association with PAOD. Our results indicated that the risk of PAOD was found to be higher in the glaucoma group than in the non-glaucoma group. Additionally, the glaucoma group had a higher risk of PAOD in the 20 to 39 age group. While part of the pathophysiology of glaucoma is known to be related to vascular changes and tissue stress response, endothelium damage may also occur in other vessels, which increases the risks of PAOD. Glaucoma is inherently associated with fragile blood vessels [24]. Glaucoma occurring before the age of 40 is known as juvenile open-angle glaucoma, which tends to have more severe symptoms compared with adult-onset primary open-angle glaucoma that occurs after the age of 40 [25,26]. Both types can lead to significant vascular damage in the optic nerve [27]. Therefore, it is possible that glaucoma associated with genetic defects and accompanied by vascular damage may result in the development of peripheral artery occlusive disease (PAOD) at a younger age. However, further research is needed to substantiate this viewpoint. Hypertensive and ischemic heart disease were associated with an increased risk of peripheral arterial occlusive disease (PAOD). In the stratified analysis conducted in this study, the glaucoma subgroup did not show statistically significant results in the two subgroups analyzed. This indicates that, when assessing the risk of PAOD, the influence of glaucoma may be lower compared with the risk posed by underlying stratified diseases. Therefore, the study revealed a significant association between glaucoma and PAOD in populations without hypertension and ischemic heart disease.

Glaucoma is a multi-tissue disease, involving the trabecular meshwork, the optic nerve head, and the visual cortex. Blood supply of the optic nerve head is important for the functional and metabolic demands of the retina. The association between retinal vascular changes with glaucoma has been shown in previous studies [28,29]. Evidence of a decreasing retinal vessel caliber with increasing glaucoma risk was demonstrated, with a stronger correlation for arteries than veins [30]. J. B. Jonas et al. evaluated the vessel diameter in normal and glaucoma eyes; the vessel diameters were significantly narrower in the glaucomatous eyes. The differences were most significant in the inferior temporal retinal artery, followed by the superior temporal artery, then the inferior temporal vein, and finally, the superior temporal vein [31]. Altered systemic vasoreactivity with endothelial cell dysfunction was also confirmed in normal-pressure glaucoma (NPG) patients [32]. Moreover, some publications had pointed out the correlation between glaucoma and retinal vein occlusion, and elevated intraocular pressure (IOP) may compress blood vessels and induce subsequent intimal hyperplasia, leading to the collapse of retinal vessel walls. Optic disc depression due to glaucoma may distort retinal vessels at the optic disc, which may predispose the vein to occlusion, leading to RVO [33,34]. For glaucoma related to retinal arterial occlusive, most central retinal artery occlusion is blood clots floating from other places, or caused by systemic inflammatory disease, which is different from the sensitivity of blood vessels caused by glaucoma. Pathological and clinical evidence of the two diseases is needed to clarify this relationship through further studies. Our study showed that the risk of PAOD for patients in the glaucoma group was higher, which may be influenced by blood flow. Regarding the relationship between ocular diseases and blood flow, there are still differences. Su et al. demonstrated impaired blood-flow-mediated vasodilation in patients with normal-tension glaucoma (NTG), which could be attributed to peripheral vascular endothelial dysfunction [35]. However, there have been differing views on the relationship between eye diseases and peripheral vascular function. A cross-sectional case–control study from 2012 to 2013 indicated no difference in microcirculation between NTG patients and controls [36]. Our study revealed that the glaucoma group had a higher risk on PAOD patients, which may be influenced by blood flow. Further studies are needed to clarify this relationship.

On the molecular level, the pathogenesis of glaucoma apparently involves the same cell adhesion molecule (CAM) that is implicated in the development of vascular diseases [37]. Scientists found that endothelial leukocyte adhesion molecule-1 (ELAM-1) is present on trabecular meshwork (TM) cells in the outflow pathways of eyes with glaucoma of diverse etiology, regardless of glaucoma subtype or severity, but is not detected in healthy eyes [38]. ELAM-1 is a cell surface glycoprotein that mediates the adhesion of blood neutrophils [39], and is also the earliest marker for atherosclerotic plaque in the vasculature. Previous studies have revealed that the expression of ELAM-1 is not related to inflammation, but is associated with the interleukin-1 (IL-1) autocrine feedback loop through transcription factor NF-kappa B [38]. Vessel endothelial injury with a dysfunction of PAOD has been reported. The decrease in vessel diameter is associated with stenosis and PAOD [40]. Previous study revealed that an elevated level of homocysteine was also a predisposing factor for atherosclerosis through damage to the vascular tissue, and was the cause of endothelial dysfunction in patients with PAOD [41]. Furthermore, the association between glaucoma and PAOD may be due to existing risk factors such as hypertension, diabetes, hyperlipidemia, cardiovascular disease, or smoking [42,43,44]. Further medical research is needed to clarify the mechanisms between glaucoma and PAOD.

There are several limitations to this study. Firstly, the database used in this study did not provide details regarding the specific type of glaucoma or the treatment protocol administered to patients, both of which could potentially influence the extent of vessel damage observed. Secondly, the database did not include information on health-related behaviors, such as diet and physical activity, which may also play a role in the development and progression of glaucoma. Thirdly, the retrospective cohort design employed in this study precludes the establishment of association relationships between variables. Lastly, as a result of propensity score matching, the reduction in the non-glaucoma group may have introduced selection bias into the study.

## 5. Conclusions

In this study, patients with glaucoma were associated with a higher risk of PAOD. Moreover, the glaucoma group had a higher risk of developing PAOD in the 20 to 39 age group.

## Figures and Tables

**Table 1:** Demographic characteristics of glaucoma and non-glaucoma patients.

Variables	Before PSM Matching		ASD	After PSM Matching		ASD
	Non-Glaucoma (N = 382,300)	Glaucoma (N = 95,575)		Non-Glaucoma (N = 95,575)	Glaucoma (N = 95,575)	
Age			<0.001			<0.001
20–39	73,832 (19.31)	18,458 (19.31)		18,200 (19.04)	18,458 (19.31)	
40–64	187,872 (49.14)	46,968 (49.14)		47,023 (49.20)	46,968 (49.14)	
≥65	120,596 (31.54)	30,149 (31.54)		30,352 (31.76)	30,149 (31.54)	
Mean ± SD	55.03 ± 16.46	55.03 ± 16.46	<0.001	55.50 ± 16.38	55.03 ± 16.46	0.029
Sex			<0.001			<0.001
Female	199,944 (52.30)	49,986 (52.30)		49,967 (52.28)	49,986 (52.30)	
Male	182,356 (47.70)	45,589 (47.70)		45,608 (47.72)	45,589 (47.70)	
Hypertension	85,750 (22.43)	27,732 (29.02)	0.151	27,849 (29.14)	27,732 (29.02)	0.003
Hyperlipidemia	38,067 (9.96)	14,765 (15.45)	0.165	14,785 (15.47)	14,765 (15.45)	0.001
Chronic liver disease	127,74 (3.34)	4491 (4.70)	0.069	4563 (4.77)	4491 (4.70)	0.004
Chronic kidney disease	4579 (1.20)	1774 (1.86)	0.054	1784 (1.87)	1774 (1.86)	0.001
Diabetes	38,885 (10.17)	17,191 (17.99)	0.226	17,162 (17.96)	17,191 (17.99)	0.001
COPD	9727 (2.54)	2885 (3.02)	0.029	3014 (3.15)	2885 (3.02)	0.008
Ischemic heart disease	21,705 (5.68)	7437 (7.78)	0.084	7570 (7.92)	7437 (7.78)	0.005
Stroke	14,063 (3.68)	4102 (4.29)	0.031	4160 (4.35)	4102 (4.29)	0.003
Intracranial bleeding	1653 (0.43)	461 (0.48)	0.007	445 (0.47)	461 (0.48)	0.002
Deep vein thrombosis	219 (0.06)	77 (0.08)	0.009	73 (0.08)	77 (0.08)	0.001
Varicose veins of lower extremities	356 (0.09)	115 (0.12)	0.008	106 (0.11)	115 (0.12)	0.003
Psoriasis	878 (0.23)	328 (0.34)	0.021	331 (0.35)	328 (0.34)	0.001

PSM: propensity score matching; ASD: absolute standardized differences; COPD: chronic obstruction pulmonary disease.

**Table 2:** Poisson regression of the relative risk of glaucoma and non-glaucoma.

Variables	Non-Glaucoma	Glaucoma
N	95,575	95,575
Person-years	708,271	724,114
No. of PAOD	2423	2994
ID (95% C.I.)	3.42 (3.29–3.56)	4.13 (3.99–4.29)
Relative risk (95% C.I.)	Reference	1.21 (1.15–1.28)

ID: incidence density (per 1000 person-years); C.I.: confidence interval; PAOD: peripheral arterial occlusion disease.

**Table 3:** Cox proportional hazard model analysis for the risk of PAOD.

Variables	Univariable	*p* Value	Multivariable †	*p* Value
	HR (95% C.I.)		HR (95% C.I.)	
Group				
Non-glaucoma	Reference		Reference	
Glaucoma	1.21 (1.15–1.28)	<0.0001	1.18 (1.12–1.25)	<0.0001
Age				
20–39	Reference		Reference	
40–64	6.21 (5.27–7.32)	<0.0001	4.75 (4.03–5.61)	<0.0001
≥65	15.45 (13.12–18.18)	<0.0001	9.32 (7.88–11.01)	<0.0001
Sex				
Female	Reference		Reference	
Male	1.00 (0.94–1.05)	0.883	1.02 (0.97–1.08)	0.491
Hypertension	2.95 (2.80–3.11)	<0.0001	1.37 (1.29–1.45)	<0.0001
Hyperlipidemia	1.84 (1.72–1.96)	<0.0001	0.89 (0.83–0.95)	<0.001
Chronic liver disease	1.44 (1.29–1.60)	<0.0001	1.06 (0.95–1.18)	0.332
Chronic kidney disease	6.23 (5.59–6.95)	<0.0001	3.36 (3.01–3.76)	<0.0001
Diabetes	3.49 (3.31–3.69)	<0.0001	2.22 (2.09–2.36)	<0.0001
COPD	2.34 (2.08–2.62)	<0.0001	1.27 (1.13–1.42)	<0.0001
Ischemic heart disease	2.67 (2.48–2.86)	<0.0001	1.31 (1.22–1.41)	<0.0001
Stroke	2.77 (2.53–3.04)	<0.0001	1.35 (1.23–1.49)	<0.0001
Intracranial bleeding	1.41 (0.97–2.04)	0.070	0.79 (0.54–1.14)	0.207
Deep vein thrombosis	5.57 (3.41–9.08)	<0.0001	2.74 (1.68–4.48)	<0.0001
Varicose veins of lower extremities	1.84 (0.99–3.43)	0.053	1.15 (0.62–2.14)	0.656
Psoriasis	1.14 (0.72–1.82)	0.572	0.91 (0.57–1.45)	0.690

PAOD: peripheral arterial occlusion disease; COPD: chronic obstructive pulmonary disease. † Adjusted for all variables.

## Data Availability

Due to the policy of the National Health Insurance Administration in Taiwan, the raw data of this study are not available.
